# 732. Sensitivity and Specificity of Point of Care Lung Ultrasound vs. Chest X-Ray for the Diagnosis of Pediatric Pneumonia in Limited resource settings: The Zambia Experience

**DOI:** 10.1093/ofid/ofab466.929

**Published:** 2021-12-04

**Authors:** Ingrid Y Camelo, Rachel Pieciak, Ilse castro-aragon, Bindu Setty, Lauren Etter, Kaihong Wang, Christopher Gill

**Affiliations:** 1 UMass- Baystate Medical Center/Baystate Children's Hospital, Northampton, Massachusetts; 2 MPH, Boston, Massachusetts; 3 Boston Medical Center, Chestnut Hill, Massachusetts; 4 Boston University, Boston, Massachusetts; 5 Boston U. School of Public Health, Boston, MA

## Abstract

**Background:**

Pediatric pneumonia is the leading cause of child mortality in low-income countries. Pneumonia diagnosis is a challenge. Chest x-ray (CXR) is considered the gold standard, but it exposes children to ionizing radiation, and access to CXR is limited to hospital settings. Lung Point of Care Ultrasound (POCUS) is a portable and non-radiating alternative to CXR.

**Methods:**

We enrolled 200 children aged 1-59 months from the University Teaching Hospital (UTH) Emergency Department (ED) in Lusaka, Zambia who met the WHO (World Health Organization) case definition for severe pneumonia. From each child, we collected demographic and clinical data, a CXR, and a set of ultrasound images using a Butterfly ultrasound probe. Images were independently interpreted by two radiologists blinded to the results of the other imaging modality. Using CXR as the gold standard, we determined the sensitivity and specificity, positive and negative predictive values, and likelihood ratios for pneumonia using lung POCUS.

**Results:**

This preliminary analysis included 50 children seen between May-October 2020. Median age (9 months) (Range 4-15). 58% were male, (29/50). Median temperature was 37.3⁰C (range 36.5-38.0); median respiratory and pulse rates were 41 breaths/min (range 31-50) and 139 beats/min (range 124-160) respectively; median SpO_2_ on RA was 91% (range 89-95). 50% of cases had difficulty breathing (82%, 41/50); chest retractions (70%, 35/50) and grunting (62%, 31/50). Ultrasound images for 49/50 (98%) cases and CXRs for 50/50 (100%) of cases we analyzed. Sensitivity of lung POCUS in the detection of CAP was 61% (95% Cl: 0.52-0.84). The specificity was 77% (95% Cl: 0.56-0.91). Positive predictive value (PPV) 70% (95% CI: 0.62-0.94) and negative predictive value (NPV) 69% (95% CI: 0.56-0.79).

**Conclusion:**

Preliminary findings of this study demonstrated the lower diagnostic accuracy of lung POCUS versus CXR in the detection of pneumonia in children 1- 59 months. The high specificity of the test will aid in ruling out severe pneumonia in children. Due to its availability, ease of interpretation, and absence of radiation exposure, lung POCUS should still be considered as an important initial imaging tool for the diagnosis of CAP in children in limited-resource settings.

**Disclosures:**

**All Authors**: No reported disclosures

